# Occupational injuries in times of labour market flexibility: the different stories of employment-secure and precarious workers

**DOI:** 10.1186/s12889-016-2834-2

**Published:** 2016-02-13

**Authors:** Massimiliano Giraudo, Antonella Bena, Roberto Leombruni, Giuseppe Costa

**Affiliations:** Department of Epidemiology, Servizio di Epidemiologia – Settore rischi e danni da lavoro – ASL TO3 – Grugliasco, Via Sabaudia 164 Grugliasco, Turin, 10095 Italy; Department of Economics and Statistics “Cognetti de Martiis”, University of Turin, Turin, Italy; Department of Clinical and Biological Sciences, University of Turin, Orbassano, Turin, Italy

**Keywords:** Occupational injuries, Flexibility, Career mobility, Work experience, Cluster analysis, Young worker, Young employee, Precarious employment

## Abstract

**Background:**

The relationship between labour market flexibility, job insecurity and occupational injuries is not univocal. The literature generally focuses on the temporary character of work arrangements rather than on the precarity of careers. The aim of this paper is to identify, without defining *a priori* what a precarious career is, the most common professional profiles of young people who entered the labour market in the 2000s and to correlate them with occupational injury risks.

**Methods:**

Using the Whip-Salute database, which combines individual work and health histories, we selected the subjects under 30 years of age whose first appearance in the database is dated after 2000. The occupational history of each individual between 2000 and 2005 was described according to 6 variables (type of entry contract, number of contracts, number of jobs, economic activities, work intensity and duration of the longest period of non-employment). Workers were grouped into homogeneous categories using cluster analysis techniques, which enable to identify different career profiles. Injury rates were calculated for each cluster, and compared within and between the groups.

**Results:**

We selected 56,760 workers in the study period, who were classified in 6 main career profiles. About 1/3 of the subjects presented an employment-secure career profile, while about 45 % of them were classified into 3 clusters showing precarious career profiles with different work intensities. Precarious workers present significantly higher injury rates than those with secure careers, with an increase in risk between 24 and 57 % (*p* < 0.05). The comparison of injury rates at the beginning and at the end of the study period revealed a significant decrease in all clusters, but the gap between secure and precarious workers remained wide.

**Conclusions:**

Cluster analysis allowed to identify career patterns with clearly different characteristics. A positive association between injury risk and the level of career fragmentation was found. The association cannot be fully interpreted in a causal way, since reversed causality and selection processes may be in action. However the study indicates a disadvantage for precarious workers, who face significantly higher risks of both minor and severe injuries.

## Background

Labour market flexibility has increased throughout the world over the past two decades. In 2012, temporary contracts made up 14.1 % of work contracts in Europe and 7.6 % in North America, comprising about 40 million individuals [1]. Following the so-called ‘Treu Package’ (law No. 196/1997) and the Biagi reform (Law Decree No. 276/2003), which introduced and regulated several new types of work arrangements, the number of temporary workers substantially increased in Italy too. The Italian National Institute of Statistics (ISTAT) estimated that in 2012 the number of temporary workers amounted to about 2.8 million, i.e., 12.3 % of all employees [2]. This phenomenon applies primarily to young people: in 2013 around 50 % of workers between 15 and 24 years of age fell into this category [3].

The introduction of flexible contracts does not necessarily have negative implications for individuals’ careers. The rationale for their introduction was to shift the attention from job security to employment security [4]: while, on the one side, flexible work arrangements are more likely to have a short duration, they also imply a higher probability of exiting unemployment, so that the net effect on the employment opportunities for individuals is ambiguous. In “*flexsecure*” systems, the potentially negative consequences of flexibility are further mitigated by the welfare state, which provides assistance to workers during their unemployment periods through social benefits, training, and support in finding new employment opportunities. The academic and public debate on the labour market functioning revolves around the concern that the balance between shorter job durations and more job openings and welfare support may result in less opportunities for individuals [5, 6]. If this is the case, flexibile work arrangements fail to contribute to an overall employment-secure career, leading instead to a series of short-duration jobs followed by sometimes long unemployment spells – a situation we will refer to as “precarity” and “precarious careers”. From an economic point of view, precarious careers in turn usually imply comparatively less opportunities for wage and career progression, smaller gains in human capital, and lower accumulation of welfare and social security contributions.

The epidemiological studies which devoted attention to the association between temporary work and occupational injuries have provided conflicting evidence. A 2005 review [7] showed that, while the number of studies with available data for effect size was not sufficient to undertake a meta-analysis, an increased risk could be identified for precarious workers in only 7 out of 13 studies. The remaining studies did not detect significant differences in occupational injury risk between precarious and non-precarious workers. A later Finnish study on temporary workers did not identify any association [8]; Italian studies are few and mostly regard interim work [9–11].

This diversity of results may be due to the fact that different studies refer to different types of temporary work – using definitions which may not be fully comparable with each other – and to different contexts depending on the country. Besides the differences, however, a limitation of current research is to be found in the static point of view implicit in most studies, when they consider as a risk factor the temporary or insecure character of a given work contract. Similarly to what we recalled about the relations between flexibility and labour market functioning, it is important to shift the focus from the temporary character of a given work arrangement to the precarity over time of an individual’s work career. This is particularly important in the assessment of occupational injury risk: it is in the case of frequent changes in duties, frequent periods of unemployment and a lower accumulation of experience that the most adverse effects on safety are to be expected.

The aim of this study is to contribute to the debate on the relationship between labour market flexibility and risk of occupational injury, by measuring the actual fragmentation and insecurity of careers in relation with injury risk. In order to do so we do not specify *a priori* what is the boundary between an employment-secure and a precarious career, but use a longitudinal dataset of work histories and health (Whip-Salute) to empirically assess what are the prevailing career paths in Italy amongst young people. These typologies will then be classified according to their level of fragmentation, and their association with different levels of injury risk will be assessed.

## Methods

### The Whip-Salute dataset

The Work and Health Histories Italian Panel (Whip-Salute) is a database of individual work histories developed starting from the administrative archives of the National Institute for Social Security (INPS), currently covering the period 1985–2005. The reference population (about 15 million individuals) is made up of people who spent all or part of their career in Italy. A 7 % systematic sample of this population was extracted on the basis of their dates of birth. For every individual the work career was compiled, lining up all their employment periods, unemployment benefits, redundancy payments, disability indemnities and retirement. The employment periods comprise employed work, self-employed work, and subcontracted work; some professional categories, such as architects and lawyers, are not included. It covers all production sectors in manufacturing, construction and services, but excludes permanent workers in the public sector and all workers in the agricultural sector. The database comprises a great variety of demographic and employment information. The section regarding employed work is a linked employer-employee database (LEED): thanks to the link with the INPS’ Observatory on Enterprises, workers’ data are merged with information regarding the companies which employ them.

The data on occupational Injuries originate from the archives of the National Insurance Institute for Occupational Injuries (INAIL). Using the same sampling criteria used for INPS archives, we extracted episodes of occupational injuries occurred between 1994 and 2005 that led to an absence from work of more than three days, as certified by a physician (certification is always required). The linkage between the two was then implemented through an encrypted unique identifier based on the individual’s tax code; it was carried out independently by the two organisations. All activities, regardless of their complexity or depth, were conducted in accordance with Italian regulations on privacy and with the approval of the national institutes involved.

In 2013, the WHIP-Salute database has been included, under the responsibility of the Ministry of Health, in the National Statistics Program that establishes what are the statistical surveys of public interest. Our institution participates in the program of development of the database, so the date that we used was openly available to us. In other cases, the Ministry of Health releases microdata files for research purposes, upon request based on a research protocol and after authorization of the Italian Data Protection Authority.

For a more detailed description of the WHIP-Salute database see Bena et al. [12].

### Statistical analysis

We selected all individuals aged 30 years or younger, who have been recorded in the Whip database for the first time in 2000 as employed, self-employed or subcontracted. We selected only young people under 30 because in Italy work precarity mainly affects this category of people.

For each individual we summarized the career over the 6-year period between 2000 and 2005 (the most recent year for which the database is currently available) according to the following 6 variables:

1. **entry contract**; three typologies were identified: ‘without time restraints’ (permanent employment or establishment of an autonomous activity); ‘seasonal’; and ‘with time restraints’ (temporary employment, interim employment, “training and work contracts” - in Italian, *contratti di formazione lavoro*, CFL -, internships, dependent self-employment, and VAT number registrations);
2. **number of contracts** which the individual has held in the period under study;
3. **number of jobs**; the distinction between jobs and work contracts was necessary to consider the case in which a stable job relation between an employer and an employee is realized through different contracts, for example a first contract of apprenticeship followed by a permanent contract;
4. **number of economic sectors** in which the individual has worked according to the 2-digit “ATECO 91” categories, which is the Italian version of Nace rev. 1;
5. **work intensity** measured as the ratio between number of months worked and number of months observed (calculated as the number of months from the first record in WHIP and December 2005);
6. **duration of the longest period of non-employment** within the 6 years under study.

The variables have been selected to represent some of the factors held as most important in the literature. As regards *entry contracts*, several studies [13, 14] have inquired on whether, for young people, entering the job market with a temporary contract represents a port of entry to stable employment or, rather, a sort of a trap. The *number of contracts* and *the number of jobs* capture the main aspect of flexibility, i.e., the frequency of job changes. It is of acknowledged relevance for the risk of occupational injuries: the more frequent job changes occur, the shorter will be the average job duration; a short job duration, in turn, is a risk factor for injuries [15–17]. The *number of switches between economic sectors* implies a loss of industry-specific human capital [18], which also has been recognized as a protective factor against occupational injuries [17]. W*ork intensity* measures the extent to which job changes are associated with periods of unemployment or non-employment. Low work intensity and long periods of non-employment are likely to put pressure on the worker to accept relatively more risky job offers, such as those offloaded by a larger organization or refused by permanent workers [19, 20] Hence, this variable allows measuring whether flexible work arrangements actually offer a wider scope of work opportunities or, rather determine a more fragile career.

The individuals under study were grouped into homogeneous categories using cluster analysis, which enabled the classification of work histories on the basis of the 6 variables described above without further *a priori* assumptions [21]. Cluster analysis is a multivariate analysis technique that allows bundling statistical units (in this case workers) into clusters that are internally homogeneous and heterogeneous between them. With this purpose, we calculated Euclidean distance indexes between pairs of observations. We applied the method of hierarchical clustering, in which each individual initially represents a cluster of its own. Then clusters are repeatedly merged until the desired cluster structure is obtained. The merging of clusters was performed according to the “average-link clustering method” [22]. The ideal number of groups was defined on the basis of the R^2^ value (the ratio of the variance explained by the partition under consideration) equalling 95 %. This choice was based on a compromise between the need for synthesis and the need for homogeneity within each group.

The analysis of the risk of injury was carried out on employed workers only, excluding the self-employed. The time at risk was measured as the number of months of actual work, which was obtained by subtracting all periods of sick and redundancy leaves from the number of paid months. In order to evaluate whether the injury risk change over time between the clusters, and within the same clusters, we split the 6-year period into two halves. For each cluster, the injury rate per 100 workers/year was calculated (confidence interval at 95 %) over the whole period, and in each triennial period. We performed our analyses on both the total number of injuries and on the number of serious injuries only (defined as those with a prognosis of over 29 days and/or permanent or fatal injury). Injury rates were compared with the Byar’s approximation [23], which gives accurate approximations of the exact Poisson probabilities.

## Results

The sample is composed of 56,760 individuals, corresponding to around 864,000 young people in the general population. The distribution of the basic demographic characteristics (Table 1) shows a greater proportion of men; the mean age of entry in the labour market is 23 years (the modal age is 20 years). Over half of the young people selected entered the labour market through a contract ‘with time restraints’. Among these, the “training and work” (CFL) and apprenticeships contracts, which are used by employers to train young persons through phases of practical and professional training, represent 25 % of the entry contracts in the study. On the other hand, two out of five young people start their career ‘without time restraints’ and one out of three with a permanent contract of employment.

**Table 1:** Characteristics of selected study subjects

			Number	Percent
Gender	Male		35,598	59.6
	Female		24,162	40.4
Entry Contract	Without time restraints		24,567	41.1
	Of which			
		Permanent contract	20,112	33.7
		Freelance activity	944	1.6
		Freelance commercial activity	1,996	3.3
	Seasonal		1,515	2.5
	With time restraints		32,193	53.9
	Of which			
		Apprenticeship	11,921	19.9
		Temporary Contract	9,833	16.5
		Dependent Self-Employment	4,340	7.3
		CFL	3,069	5.1
		Interim	2,687	4.5
		VAT registration	343	0.6
Number of contracts		1	12,280	20.5
		2	12,712	21.3
		3	10,626	17.8
		4	7,730	12.9
		5	5,069	8.5
		>5	8,343	14.0
Number of jobs		1	15,944	26.7
		2	12,975	21.7
		3	9,488	15.9
		4	6,713	11.2
		5	4,414	7.4
		>5	7,226	12.1
Number of Economic Activities (Sectors)		Not Stated	5,021	8.4
		1	27,615	46.2
		2	15,063	25.2
		3	6,336	10.6
		4	2,021	3.4
		>4	704	1.2
Work intensity (percentage)		0–25 %	11,222	19.8
		25–50 %	7,737	13.6
		50–75 %	9,389	16.5
		75–100 %	28,412	50.1
Duration of longest period of non-employment		<6 months	21,184	37.3
		6–11 months	9,180	16.2
		12–23 months	8,621	15.2
		24–35 months	5,502	9.7
		> = 3 years	12,273	21.6

About 40 % of the young people in our sample have relatively stable careers (corresponding to 2 work contracts over 6 years); instead, a relevant share of the individuals analyzed (14 %) shows significant career fragmentation, with more than 5 work contracts in 6 years. The distribution of work episodes substantially matches that of work contracts.

Around 45 % of the individuals in our sample work within a single sector over the 6 years under study, and 25 % in 2 sectors. As to work intensity, on average, the subjects worked about 65 % of the period under observation, with, however, a rather high standard deviation (34 %).

The distribution of the longest non-employment duration is highly polarised. Around a quarter of the subjects is never non employed, and around a quarter had a longest period of non-employment lasting up to 1 year. Among these, there are young people who present significant periods of unemployment within the 6 years covered by the analysis: around 15 % present a gap in employment of over 4 years.

**Fig. 1:**
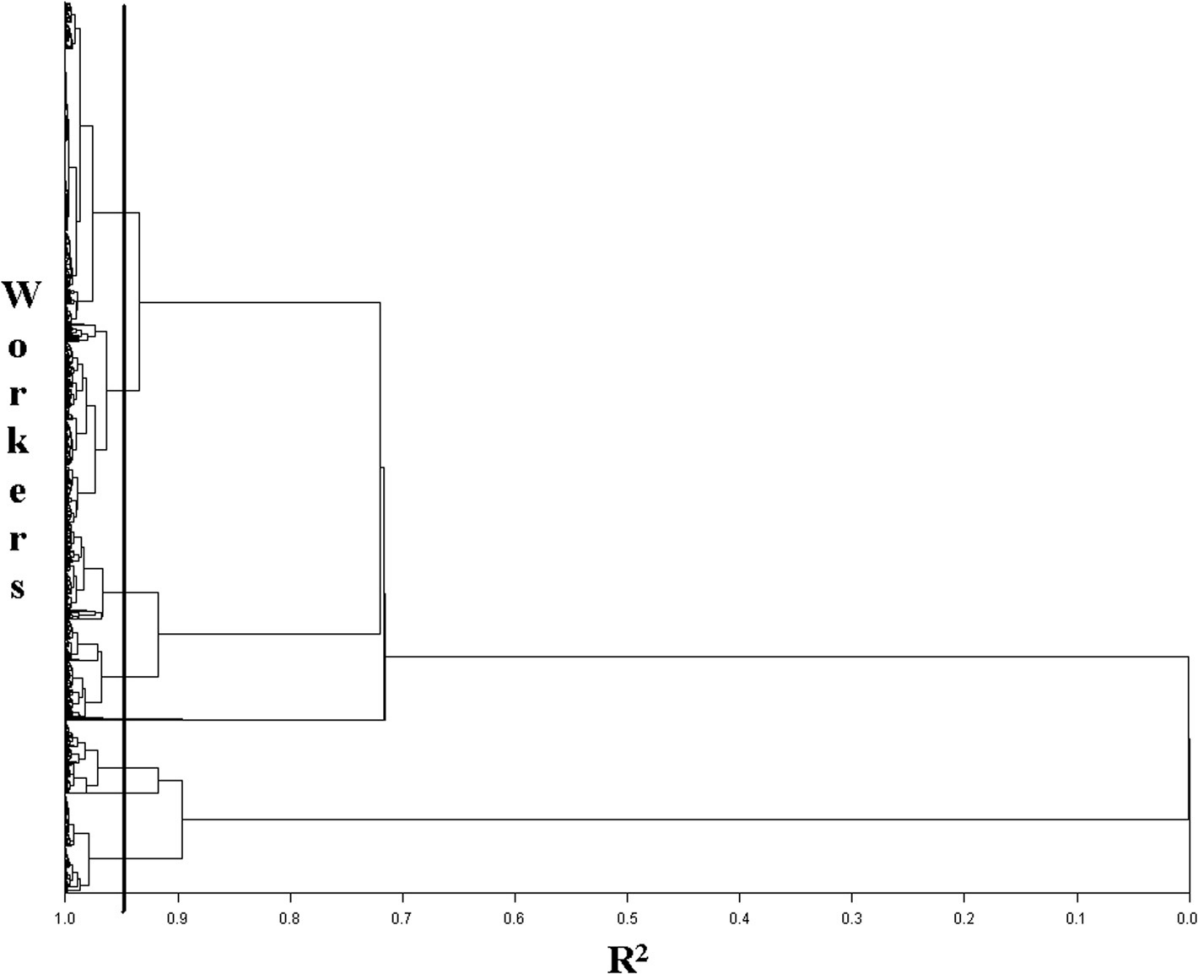
Dendrogram representing the nested grouping of workers and similarity levels at which groupings change

A standard way of displaying the results of the hierarchical clustering method is the dendrogram (Fig. 1), representing with a tree structure the nested grouping of workers. The higher the level of aggregation of individuals, the lower is the internal homogeneity of the groups and the heterogeneity between groups. With a R^2^ value of 95 %, the *cluster analysis* technique led to grouping the workers in 16 categories, the first 6 of which include 99 % of subjects, while the remaining 10 categories comprise outlier cases, *i.e.,* individuals with extreme characteristics. Looking at the groups’ characteristics (Table 2) it is possible to describe the main career profiles identified (every cluster is given a title which summarises its main characteristics):

**Table 2:** Average characteristics of individuals in each cluster

CLUSTER	Number	%	Entry contract (in %)			Number of contracts	Number of jobs	Number of economic activities	Work intensity (in %)	Duration of longest period of non-employment (in months)
			Without time restraints	Seasonal	With time restraints					
1	20,478	36.1	44	1	55	3	2.6	1.4	98	1
2	6,323	11.1	40	4	55	1.2	1.1	0.9	7	62
3	4,627	8.2	41	4	55	2.1	2	1.3	22	48
4	6,263	11	39	4	57	3	2.9	1.6	36	33
5	6,176	10.9	36	4	60	4.2	4	1.9	53	21
6	12,709	22.4	38	3	59	4.8	4.5	2	75	10
7	79	0.1	20	3	77	17	16.6	3	80	7
8	43	0.1	26	5	70	23.4	23.1	2	77	7
9	30	0.1	20	3	77	16.9	16.9	1.7	46	23
10	11	0	9	0	91	34.4	34.3	2.3	79	7
11	7	0	14	0	86	30.9	30.7	1.7	61	15
12	6	0	33	0	67	10.3	10.3	2.2	28	44
13	3	0	0	0	100	42.3	42.3	1.7	90	3
14	2	0	0	0	100	29	29	1	54	30
15	2	0	0	0	100	44	44	1	68	18
16	1	0	0	0	100	82	81	3	96	1

- Cluster 1: individuals with an employment-secure career.
  This is the largest group (36 % of subjects). The individuals in this group present the greatest proportion of entry contracts without time restraints, have on average 2.6 work episodes within a single economic sector and almost no interruptions between them (work intensity equal to 98 %).
- Cluster 2: individuals exiting the labour market within the first year.
  The second group comprises young people who had a single short work episode in 2000 and have then exited the labour market.
- Cluster 3: individuals exiting the labour market in the second year.
  The third cluster includes individuals with a career made of, on average, 2 work episodes within a single economic sector, but with low work intensity (22 % of the observed period); these individuals exited the labour market in 2001.
- Cluster 4: individuals with a precarious career and low work intensity.
  The fourth cluster identifies an insecure career profile, made up on average by 3 work episodes in 2 different economic sectors. This cluster is marked by low work intensity: 36 % of the observed period. It is also interesting to note that the average interruption lasts around 33 months, i.e., about half of the observed period.
- Cluster 5: individuals with a precarious career and medium work intensity.
  This group includes subjects with an insecure career profile, made up of an average of 4 work episodes in 2 economic sectors. The mean work intensity of this group is higher compared to the previous cluster (around 53 % of the observed period).
- Cluster 6: individuals with a precarious career and high work intensity.
  This group is the second largest (22 % of individuals) and is made up of an average 4.5 work episodes in 2 different economic sectors. Work intensity is the highest among the precarious clusters, but still very far from the employment-secure one (75 % versus 98 %).

Overall, individuals with a precarious career account for 44 % of the study group.

Table 3 presents the rate and the incidence rate ratio (IRR) in the observed period for all injuries and serious ones in the 6 main clusters.

**Table 3:** Number of injuries, rate of all injuries and serious ones (CI 95 %) and incidence rate ratio (CI 95 %) in the clusters, 2000–2005

Cluster	Profile	All injuries			Serious injuries		
		Number of injuries	Rate of injuries (100persons/year)	IRR	Number of injuries	Rate of injuries (100persons/year)	IRR
1	Employment-secure career	3671	4.39	1.00	604	0.72	1.00
			(4.25–4.53)			(0.66–0.78)	
2	Exiting the labour market in the 1st year	64	6.42	1.46	15	1.50	2.08
			(4.85–7.99)	(1.14–1.87)		(0.74–2.27)	(1.25–3.48)
3	Exiting the labour market in the 2nd year	164	5.33	1.22	45	1.46	2.03
			(4.52–6.15)	(1.04–1.42)		(1.04–1.89)	(1.50–2.75)
4	Precarious career – low work intensity	415	5.45	1.24	83	1.09	1.51
			(4.93–5.98)	(1.12–1.38)		(0.86–1.32)	(1.20–1.90)
5	Precarious career – medium work intensity	778	6.89	1.57	135	1.20	1.67
			(6.41–7.38)	(1.45–1.70)		(0.99–1.40)	(1.38–2.00)
6	Precarious career – high work intensity	2294	6.17	1.41	380	1.02	1.42
			(5.92–6.42)	(1.34–1.48)		(0.92–1.13)	(1.25–1.61)

The first group, which includes individuals with a flexible but employment-secure career, presents the lowest risk of injury compared to all other clusters. In the second and third groups, which include young people who early exited the labour market, IRR is higher by 46 and 22 % respectively. Compared with workers in the employment-secure cluster, individuals with precarious careers (clusters 4, 5 and 6) are also facing significantly higher risks: the increase in IRR ranges between 24 and 57 %. A comparison of the injury rates in clusters 5 and 6 shows that the difference between the two groups is barely significant. The same pattern applies to serious injuries; however, the increase in risk associated with less stable work careers is always greater for serious injuries than for all injuries.

**Fig. 2:**
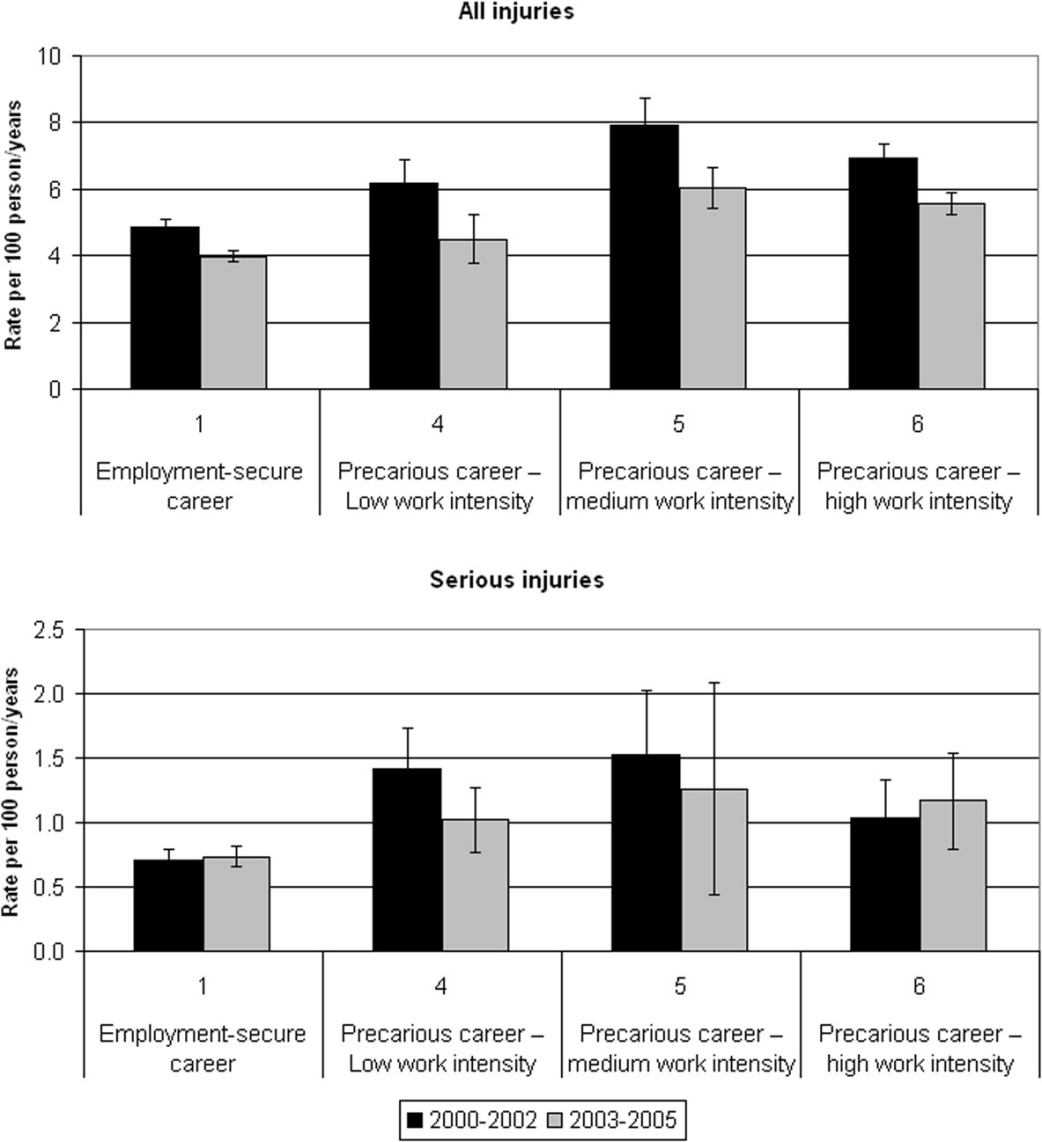
Comparison between rate of all injuries and rate of serious ones for each cluster between 2000–2002 and 2003–2005 (clusters 2 and 3 excluded from the analysis)

Figure 2 presents the injury rates for each cluster in each triennial period. Clusters 2 and 3 are not included as they comprise individuals who exited the labour market and hence were not part of the study population in the second 3-year period. In the four clusters considered in the analysis, the risk for all injuries decreases significantly in the second period. Individuals with a precarious career and medium/high work intensity (clusters 5 and 6), despite the decrease over time, retain a risk which is significantly higher than that for employment-secure workers (cluster 1). The analysis of serious injuries shows no significant difference within each cluster between the 2 periods.

## Discussion

Much of the literature on the relations between labour market flexibility and occupational injuries considered the temporary character of the work contract held by individuals at a given point of time as a risk factor. Work precarity however should be considered as a longitudinal feature of work careers, since temporary contracts may be the building blocks both of flexible but employment-secure careers and of fragmented and insecure ones. To gain a wider view on the relations between labour market flexibility and injury risks, we summarized the actual evolution of young people careers over the first six years after their labour market entry.

The analysis confirms the impact of flexibilization policies on the work careers of young people. More than 50 % of them entered our study population first time in 2000 with a temporary contract and, over the 6 years under study, 1 individual out of 3 had 4 or more work contracts (Table 1). We distinguished between work contracts and work episodes drawing on the hypothesis that these two concepts express different aspects of a work history; however, the analysis of their distribution shows no major differences between them.

The analysis of economic sectors shows that mobility between jobs often implies shifting sectors: 15 % of youth has worked in 3 or more different economic sectors. Career fragmentation also determines periods of inactivity which can be very long: around 30 % of workers present a work interruption of over 2 years.

The main aspects of flexibility, summarized in six variables representing some of the factors held as most important in the thematic literature, were jointly considered using Cluster analysis, without *a priori* assumptions on how they combine into the definition of work precarity. This allowed for the first time, to our knowledge, to classify individuals not based on the type of contract, but looking at the actual evolution of work careers.

The Cluster analysis identified 6 main groups (Table 2), which altogether represent 99 % of the young workers in our sample. The remaining ones are ‘outliers’, i.e., young people who present peculiar characteristics and who, for this reason, were excluded from the analysis. The dendrogram in Fig. 1 graphically illustrates the sequence of aggregation of individuals into homogeneous groups.

A closer inspection of the clusters revealed the presence of 3 main career profiles. The first is made up of employment-secure workers and includes 36 % of subjects. The second identifies young people who exited the labour market after a brief period of work: a phenomenon which is frequent in Italy and already highlighted in [24].

The third profile is composed of young people with a precariuos career and different levels of work intensity (clusters 4-5-6). Such group is the largest one (44 % of individuals), which confirms the idea that for a significant part of the young population the flexibilization of the labour market turned into careers with a high level of precarity.

The analysis of injury risks showed that, throughout the observed period, young people with an employment-secure career profile present lower risks compared to the ones in the other clusters (Table 3). In particular, individuals within the precarious clusters face significantly higher injury rates than those with employment-secure careers.

The difference between groups can be explained by various factors already highlighted in the existing literature. There may be a difference in working conditions, which are more dangerous for temporary workers [25], and there may be less or no safety training for those with a temporary contract compared to those with a permanent one [26]. The longitudinal view on careers helps reconciling the large number of studies which found an association between temporary work and risk of injuries with those in which such an association is not found, once adjusting for the duration and other characteristics of work [8]. The main causal links acting over time can be identified in the accumulation of work experience. Newly-hired workers present a higher risk of injury due to low familiarity with work duties, while as job-specific experience is accumulated the risks tend to decrease [27]. A protective role is also played by sector-specific experience gained in previous jobs with similar duties, but such a protection is only partial [17]. Individuals with a precarious career (Clusters 4-5-6), then, face a higher risk due to two effects. Since they have jobs of a lower average duration, they have on average a lower job-specific experience; since they more often switch across economic sectors, they benefit of a lower protective effect from the human capital accumulated in previous jobs.

It can be suggested that a further factor differentiating the individuals in the precarious clusters with respect to those belonging to the secure one is the urge of re-entering the labour market. After a job loss, if a subject faces a low pressure to get a new job – for instance because of accrued savings or for the support received from the welfare – s/he may evaluate various employment opportunities, undertake a training period, and turn down lower-paid or higher-risk jobs. On the other hand, individuals with greater need for employment will not be able to choose which job to take on and will tend to accept any opportunity. Since social security rights and individual savings are proportional to how much an individual has been able to stay in employment, this is another pathway which goes from lower work intensity to higher risks.

It must be highlighted that the association presented in this paper should not be understood as strictly causal. There is a possible issue of selection, linked to the fact that lower-skilled individuals may have at the same time a greater probability of injury and a lower probability of finding a stable occupation. In fact, the differences between clusters noted in the first 3-year period are already of great magnitude, suggesting that there may be systematic differences between workers already at their labour market entry.

There may also be an issue of reversed causality, meaning that suffering more injuries may hinder people’s ability to achieve a stable career. A comparison between career types, such as the one presented here, does not allow identifying the direction of the causal link between career profiles and injury risk. Only a longitudinal study would enable the identification of the direction of this association. Evaluating if, and how, the risk among workers has changed over time by type of career may be an indicator of how experience, however gained (either within the same contract for permanent workers, or through more work episodes for temporary ones), may affect this phenomenon. A first indication that such dynamics may be at play, although limited, is offered in our comparison of injury rates across the first and second triennial periods (Fig. 2). The analysis demonstrates a clear decrease in risks within each cluster, confirming the protective effect of accumulated experience, as already noted in the literature. However, even at the end of the period, greater risks are observed among precarious workers with medium/high work intensity (clusters 5–6) than in workers with employment-secure careers (cluster 1). It could be argued that, while greater experience does reduce the risk among precarious workers, the loss in human capital associated with frequent job changes remains a risk factor even by more experienced workers.

Regarding the rates of serious injury, there are no significant differences within each cluster between the first and second 3-year period. Hence, work experience shows a protective role mainly against minor injuries; as to severe ones, it might be the case that the current regulations and practices are effective also for newly hired workers. To our knowledge, the literature has not addressed the different impact of experience on minor vs. severe injuries, a distinction which would deserve closer investigation.

This study has some methodological limitations mainly due to data sources. Because the data on injuries stem from the official INAIL records, it is possible that injury events are under-reported: as highlighted in the literature, the fear of losing a job may induce people not to report the injury event to the insurance agency, thus causing an underestimation of risks [28]. This phenomenon tends to relate more to temporary workers who face a higher risk of not having their contract renewed, thus the real difference in risk between permanent and temporary workers is likely to be higher than the one reported here. The analysis of two health outcomes enables to control for this phenomenon, since serious injuries are considered a more valid measure of risk. In fact, the risk of serious injury (Table 3) is higher than that of all injuries: this may be interpreted as an indication of under-reporting. The issue of under-reporting had also implications for the selection of our sample: considering the high under-reporting of events in freelance and subcontracted work (dependent self employment and VAT registered) we chose to focus our analyses on employed workers only.

Another issue linked to the nature of the data relates to the fact that some types of work episodes are not recorded in INPS’ archives, namely permanent public sector workers, workers in the agricultural sector and certain high-profile professional categories. In this study, we name ‘interruptions of work’ all periods during which we do not observe work episodes. These may correspond to different situations: the worker may be unemployed (looking for work), inactive (not looking for work), working irregularly, or engaged in one of the above-mentioned unrecorded sectors. However, in our analysis we mainly focus on individuals who after a work interruption are observed again in the data. Since the chances for a permanent worker in the public sector or a high-level professional to return to the private sector as a dependent worker are extremely low, it is plain that the work interruptions that we analyze are critical times in a career, most probably periods of unemployment, discouragement or irregular work activities.

Another limitation of this study is that, while we described the temporal and contractual characteristics observed in individual work histories to identify the most frequent career profiles, we had to neglect other characteristics which may deserve further attention. One such factor is the economic one. Precarity certainly affects people’s ability to earn a living. The welfare system may have an important role in limiting the economic consequences of work precarity: among those with many periods of unemployment, individuals who qualify for unemployment benefits are less economic insecure, and thanks to this they can be more selective about work opportunities and possibly refuse higher risk duties. These aspects are not included in this study, but are under consideration for further research.

Other aspects not considered here which deserve further attention relate to the role of sectors of activity. The epidemiological literature suggests that temporary contracts concentrate in economic activities characterised by higher risks [20]. Also for this aspect, a longitudinal study could test whether it is the type of activity which is the true determinant of injury risk (and hence whether there is a work to health link), or if the less healthy subjects, who present a higher risk of injury, concentrate in certain economic activities (hence, the link is the health to work one).

## Conclusions

The study analyzed the relations between occupational injury risk and the degree of precarity of work careers among young people, who are the ones primarily involved – particularly in Italy – in the flexibilization of the labour market [3].

The results found are significant for two reasons. Firstly, from the point of view of the definition of exposure: the *cluster analysis* method enabled the classification of workers into 6 career profiles, with a clear ranking according to their level of precarity. Often, in the epidemiological or economical studies, precarious or permanent workers are identified based only on the type of contract (fixed-term contracts vs open-ended contracts), considering a single employment. In this study, for the first time, we shift from this definition of precarious work to one looking at the actual evolution of young workers’ careers.

Secondly, because of the association found between precarity and injury risk: young people with flexible but employment-secure careers present injury rates significantly lower compared to individuals for whom flexible work arrangements turn into chronically unstable, precarious careers. Within all groups, the risk of minor injuries decreases with time, but the difference between precarious and non precarious workers is significant also at the end of the study period.

The creation of categories which describe work histories has been applied in this first study from a cross-sectional point of view. This may pose a limit to the interpretation of the association between career profiles and risk of injury, since it is not possible to control for possible effects of selection or reversed causality. The use of the longitudinal dimension of the database used to address these two issues may become a further development of this work.

## Abbreviations

CI: confidence interval
INAIL: Italian Workers Compensation Authority
INPS: Italian National Social Security Institute
IRR: incidence rate ratio
WHIP-Salute: Work and Health Histories Italian Panel
